# A highly efficient transcriptome-based biosynthesis of non-ethanol chemicals in Crabtree negative *Saccharomyces cerevisiae*

**DOI:** 10.1186/s13068-023-02276-5

**Published:** 2023-03-04

**Authors:** Zhen Yao, Yufeng Guo, Huan Wang, Yun Chen, Qinhong Wang, Jens Nielsen, Zongjie Dai

**Affiliations:** 1 Tianjin Institute of Industrial Biotechnology, Chinese Academy of Sciences, Tianjin, 300308 China; 2 National Center of Technology Innovation for Synthetic Biology, Tianjin, 300308 China; 3 Laboratory of Evolutionary and Functional Genomics, School of Life Sciences, Chongqing University, Chongqing, 401331 China; 4 Department of Biology and Biological Engineering, Chalmers University of Technology, 412 96 Gothenburg, Sweden; 5 Beijing Advanced Innovation Center for Soft Matter Science and Engineering, College of Life Science and Technology, Beijing University of Chemical Technology, Beijing, 100029 China

**Keywords:** Microbial production, Crabtree negative, Chassis strain, *Saccharomyces cerevisiae*, Crabtree effect, Fatty acids

## Abstract

**Background:**

Owing to the Crabtree effect, *Saccharomyces cerevisiae* produces a large amount of ethanol in the presence of oxygen and excess glucose, leading to a loss of carbon for the biosynthesis of non-ethanol chemicals. In the present study, the potential of a newly constructed Crabtree negative *S. cerevisiae*, as a chassis cell, was explored for the biosynthesis of various non-ethanol compounds.

**Results:**

To understand the metabolic characteristics of Crabtree negative *S. cerevisiae* sZJD-28, its transcriptional profile was compared with that of Crabtree positive *S. cerevisiae* CEN.PK113-11C. The reporter GO term analysis showed that, in sZJD-28, genes associated with translational processes were down-regulated, while those related to carbon metabolism were significantly up-regulated. To verify a potential increase in carbon metabolism for the Crabtree negative strain, the production of non-ethanol chemicals, derived from different metabolic nodes, was then undertaken for both sZJD-28 and CEN.PK113-11C. At the pyruvate node, production of 2,3-butanediol and lactate in sZJD-28-based strains was remarkably higher than that of CEN.PK113-11C-based ones, representing 16.8- and 1.65-fold increase in titer, as well as 4.5-fold and 0.65-fold increase in specific titer (mg/L/OD), respectively. Similarly, for shikimate derived *p*-coumaric acid, the titer of sZJD-28-based strain was 0.68-fold higher than for CEN.PK113-11C-based one, with a 0.98-fold increase in specific titer. While farnesene and lycopene, two acetoacetyl-CoA derivatives, showed 0.21- and 1.88-fold increases in titer, respectively. From malonyl-CoA, the titer of 3-hydroxypropionate and fatty acids in sZJD-28-based strains were 0.19- and 0.76-fold higher than that of CEN.PK113-11C-based ones, respectively. In fact, yields of products also improved by the same fold due to the absence of residual glucose. Fed-batch fermentation further showed that the titer of free fatty acids in sZJD-28-based strain 28-FFA-E reached 6295.6 mg/L with a highest reported specific titer of 247.7 mg/L/OD in *S. cerevisiae*.

**Conclusions:**

Compared with CEN.PK113-11C, the Crabtree negative sZJD-28 strain displayed a significantly different transcriptional profile and obvious advantages in the biosynthesis of non-ethanol chemicals due to redirected carbon and energy sources towards metabolite biosynthesis. The findings, therefore, suggest that a Crabtree negative *S. cerevisiae* strain could be a promising chassis cell for the biosynthesis of various chemicals.

**Supplementary Information:**

The online version contains supplementary material available at 10.1186/s13068-023-02276-5.

## Background

With growing concern regarding environmental protection and energy security, microbial productions of biobased chemicals are getting increasing attention*.* Among them, *Saccharomyces cerevisiae* is a widely used model organism due to its advantages of well-understood genetic background, feasible molecular manipulation and high robustness to industrial conditions [1–4]. However, the ethanol overflow metabolism of *S. cerevisiae*, also referred to as the Crabtree effect, causes considerable loss of carbon flux in the production of non-ethanol chemicals [5].

Attempts to delete ethanol biosynthetic pathway have been, therefore, made to achieve Crabtree negative *S. cerevisiae* to eliminate the carbon loss, and in this context, since alcohol dehydrogenase (*ADH*) converts aldehyde into ethanol, deletion of all *ADH* isozymes has been envisaged as a potential solution [6]. However, ADH activity is required for the synthesis of many other specific metabolites, e.g., higher alcohols [7]. At the same time, pyruvate decarboxylase, encoded by *PDC*, converts pyruvate to aldehyde in the cytoplasm, followed by conversion to acetate, i.e., the precursor of acetyl-CoA. Hence, even though inactivation of *PDC*s genes enables redirection of metabolic flux from ethanol towards the productions of target chemicals [8], Pdc^−^ strains are notoriously known for inability to grow in the presence of excess glucose medium due to inadequate supply of acetyl-CoA as well as NAD^+^ regeneration. Furthermore, although adaptive laboratory evolution (ALE) [9] and internal deletion in a transcriptional regulator *MTH1* [10] relieved the above growth defect of Pdc^−^ strains in glucose-containing medium, the lag phase was 3 days longer and the max specific growth rates were only 0.1 h^−1^, thus impeding the strains’ industrial applications for biosynthesis.

To increase the availability of cytoplasmic acetyl-CoA for Pdc^−^ strains, pyruvate dehydrogenase complex (*PDH*), responsible for acetyl-CoA synthesis in mitochondria, was relocated to the cytoplasm [11]. However, the specific growth rate remained low due to PDH’s complex need for cofactors, one of which (lipoic acid) was absent in the cytoplasm. Consequently, the Pdc^−^ strains require a more efficient pathway for supplying acetyl-CoA. Recently, a heterogeneous pathway involving pyruvate oxidase (*PO*)/phosphotransacetylase (*PTA*) was used to replace the native *PDC*s, constructing a Crabtree-negative *S. cerevisiae* with alternative acetyl-CoA supplying pathway. In addition, double deletions of glycerol 3-phosphate phosphatase (*GPP1*, *GPP2*) decreased the accumulation of acetate. Finally, using ALE and reverse engineering conferred the Pdc^−^ strain with two genetic mutations, namely, *GPD1*^W71^* and MED2*^432Y^, forming strain sZJD-28 (Table 1). This strain sZJD-28 produced no ethanol and most carbon source was distributed to biomass and CO_2_ with growth rate reaching at 0.228 h^−1^ in glucose medium [12]. To our knowledge, sZJD-28 is the fastest growing Crabtree negative *S. cerevisiae*, with great prospects in industrial biotechnology. However, the comparison of transcriptional patterns between Crabtree negative sZJD-28 and Crabtree positive yeast has not been demonstrated, with the former’s biosynthesis capacity also being unclear.

**Table 1:** Strains list in this study

Strains	Genotype	
CEN.PK113-11C	MATa *MAL2-8c SUC2 ura3-52 his3Δ1*	Kötter, University of Frankfurt, Germany
CEN.PK YMZ-E1	MATa *ura3-52 his3-∆1 pdc1∆ pdc5∆ pdc6∆*	[77]
sZJD-28	CEN.PKYMZ-E1 acs2Δ::TEF1p-POav-ADH1t acs1Δ::TEF1p-PTAse-CYC1t gpp1Δ gpp2Δ X-2::KanMX-TEF1p-Cas9-CYC1t MED1*^432Y^ *GPD1*^W71^*	[12]
11C-BD	CEN.PK113-11C plasmid-pSP-GM2	This study
28-BD	sZJD-28 plasmid-pSP-GM2	This study
11C-BD-E	CEN.PK113-11C plasmid-2, 3-BD GM2	This study
28-BD-E	sZJD-28 plasmid-2, 3-BD GM2	This study
11C-LA	CEN.PK113-11C plasmid-LDH GM2	This study
28-LA	sZJD-28 plasmid -LDH GM2	This study
11C-PCA	CEN.PK113-11C *XII-2::TEF1p-TAL-CYC1t*	This study
28-PCA	sZJD-28 *XII-2::TEF1p-TAL-CYC1t*	This study
11C-Lyc	CEN.PK113-11C *XII-2::CYC1t-CrtE-pCDC19-pCCW12-CrtB-ADH1t-TDH2t-CrtI-pTDH3*	This study
28-Lyc	sZJD-28 *XII-2::CYC1t-CrtE-pCDC19-pCCW12-CrtB-ADH1t-TDH2t-CrtI-pTDH3*	This study
11C-Far	CEN.PK113-11C *XII-2::ADH1t-FS-pTEF1*	This study
28-Far	sZJD-28 *XII-2::ADH1t-FS-pTEF1*	This study
11C-HP	CEN.PK113-11C *XII-2::TEF1p-MCR-CYC1t*	This study
28-HP	sZJD-28 *XII-2::TEF1p-MCR-CYC1t*	This study
11C-FFA	CEN.PK113-11C *pox1Δ faa1Δ faa4Δ*	This study
28-FFA	sZJD-28 *pox1Δ faa1Δ faa4Δ*	This study
28-FFA-E	sZJD-28 *pox1Δ faa1Δ faa4Δpah1Δ*	This study

In the present study, analysis of Crabtree negative yeast sZJD-28 showed global transcriptional differences related to Crabtree positive strain CEN.PK113-11C, including enhanced carbon metabolism processes and down-regulated protein translation processes. To prove the increased metabolic fluxes towards biosynthesis, non-ethanol products derived from four representative metabolic nodes including pyruvate, malonyl-CoA, acetoacetyl-CoA and shikimate were also tested for both strains. The results highlighted obvious advantages of sZJD-28 in terms of the titer, yield and specific titer of the non-ethanol chemicals. Overall, the results suggested that, as a novel chassis cell, a Crabtree negative *S. cerevisiae* holds great potential for biomanufacturing of various chemicals.

## Results and discussion

### Transcriptional comparison between Crabtree negative and positive *S. cerevisiae*

**Fig. 1:**
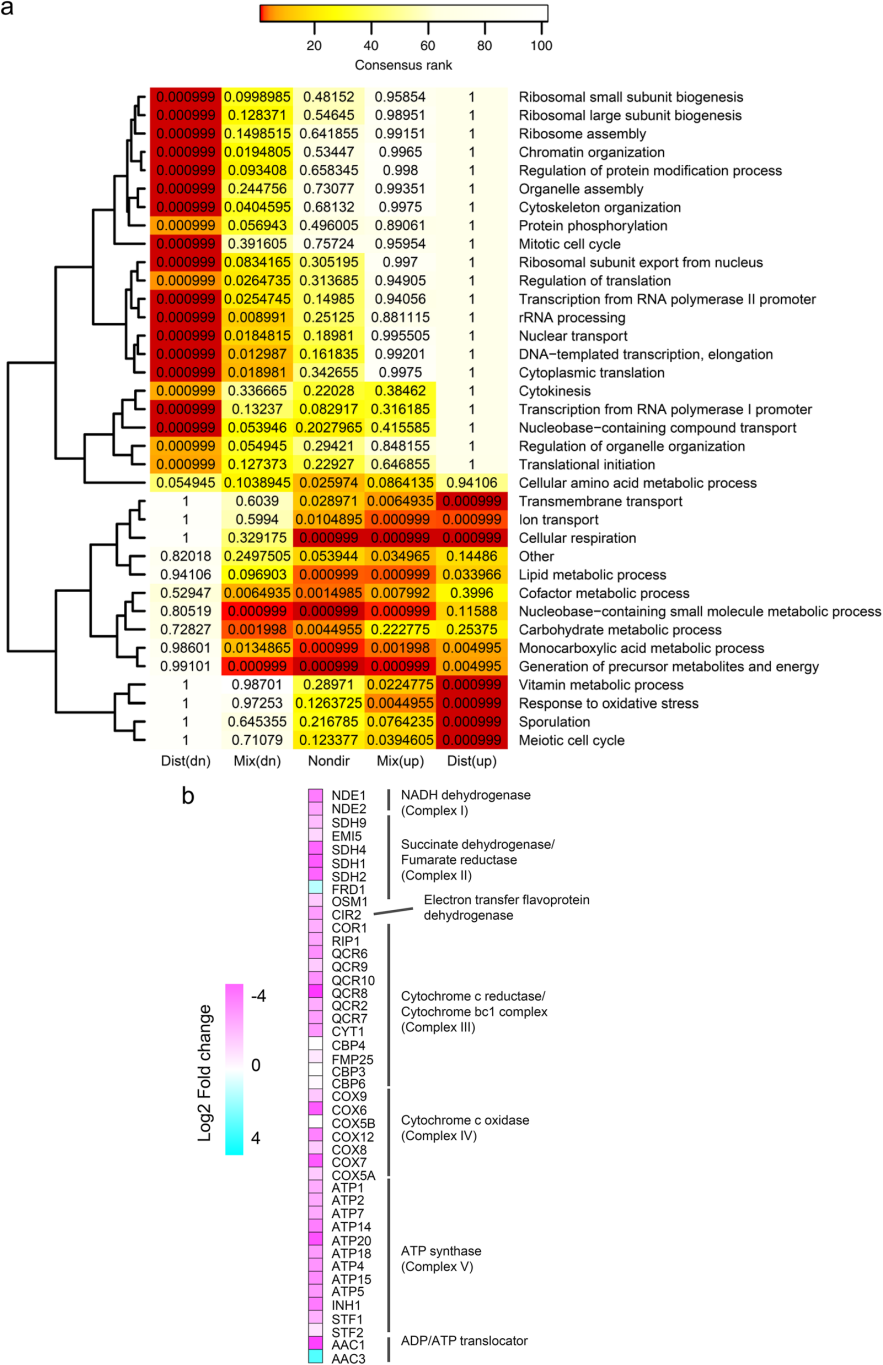

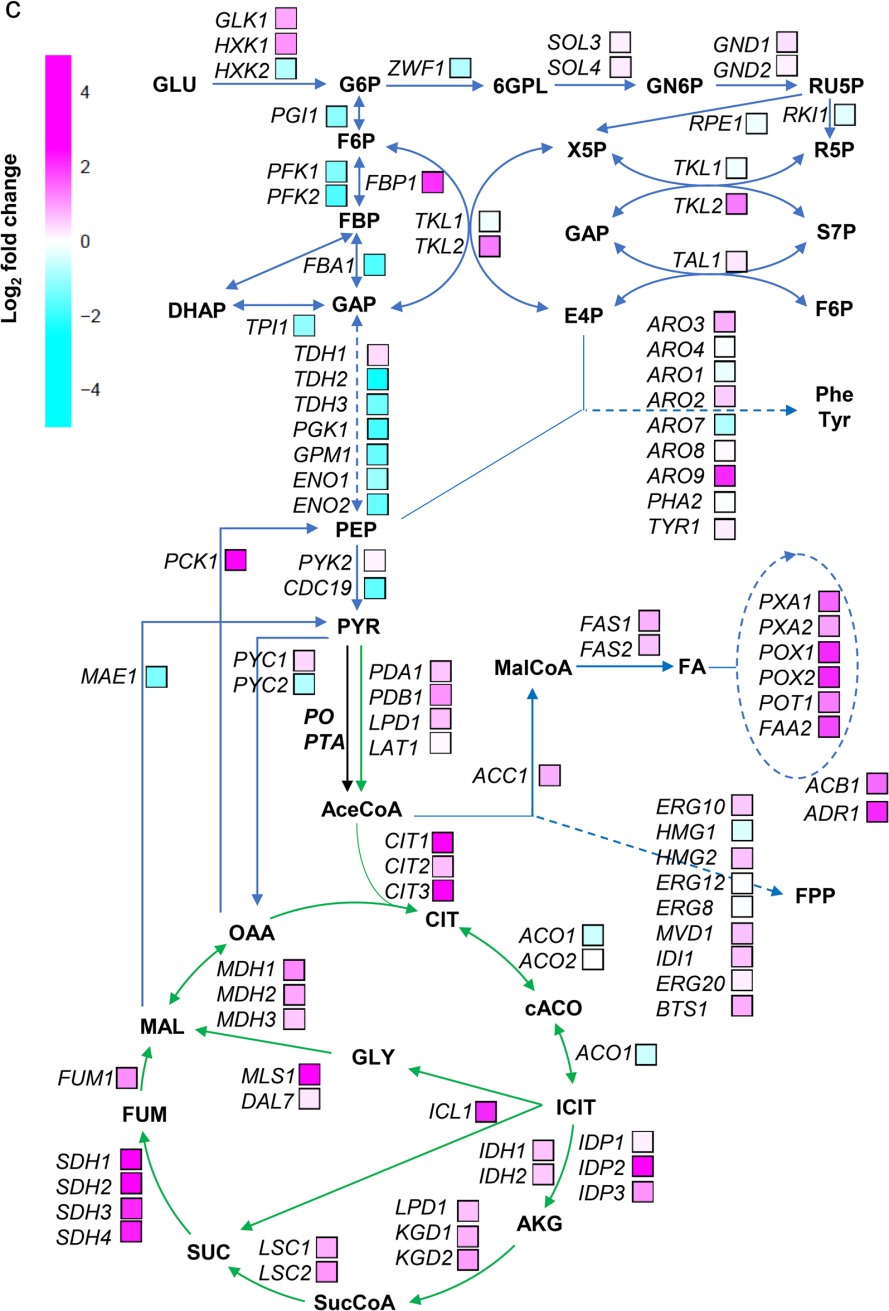
Transcriptional analysis of sZJD-28 and CEN.PK113-11C. **a** Gene sets with significant differences in sZJD-28 compared with CEN.PK113-11C. GO terms are colored according to their ranking, and each cell of the heatmap includes the significance of the GO terms. Dist (dn) is distinct-directional down, representing this gene set is coordinately down-regulated. Dist (up) is distinct-directional up, representing this gene set is coordinately up-regulated. Mix (dn) is mixed directional down and Mix (up) is mix-directional up, separately providing the significance of subsets of up- and downregulated genes in a gene set. Nondir is non-directional change, providing gene set *p* values obtained by omitting the direction of differential expression [13]. **b** Transcriptional level of genes related to oxidative phosphorylation (OXPHOS) in sZJD-28 compared with CEN.PK113-11C. **c** Schematic representation of the differential transcriptional changes of genes involved in the glycolysis, tricarboxylic acid (TCA) cycle, mevalonate (MVA) pathway, pentose phosphate pathway (PPP), fatty acid synthesis and shikimate pathway. Color keys demonstrate transcriptional fold changes in sZJD-28 relative to CEN.PK113-11C

To elucidate the mechanisms underlying the physiological differences between Crabtree positive and negative *S. cerevisiae*, the transcriptional profiles of sZJD-28 were compared with that of CEN.PK113-11C using the reporter Gene Ontology (GO) term analysis [13]. Results showed that genes associated with GO terms related to protein translation processes, such as ribosome biogenesis and assembly, rRNA processing, cytoplasmic translation, translational initiation, transcription from RNA polymerase I and II promoter, chromatin organization and mitotic cell cycle, were down-regulated in sZJD-28 (Fig. 1a). In addition, compared with the faster growing CEN.PK113-11C, the transcriptome changes related to the ribosome bioprocess of sZJD-28 further confirmed the growth law that ribosomal protein fraction are related to growth rate [14].

On the other hand, genes associated with GO terms related to cellular intermediates metabolism were up-regulated in sZJD-28. In particular, the up-regulation of genes related to cellular respiration, lipid, cofactor and carbohydrate metabolic processes, metabolites and energy generation, as well as transmembrane transport-related genes (Fig. 1a), hinted at an increase in the carbon metabolic capacity of Crabtree negative sZJD-28. Meanwhile, strengthened carbon metabolism may also cause stress responses due to the accumulation of intermediates, and this was revealed by up-regulated stress-responsive genes (Fig. 1a). Agreement with this observation, results of reporter transcriptional factors (TFs) analysis highlighted the significantly altered expression of stress-responsive TFs, such as *HSF1*, *YAP1*, *MSN2*, *CST6*, *MSN4* in sZJD-28 compared with CEN.PK113-11C (Additional file 1: Fig. S1).

The central carbon and energy metabolism which were significantly changed at transcriptional levels are described in Fig. 1b, c. In an overall view, glycolysis in sZJD-28 was down-regulated, while PP pathway, TCA cycle and oxidative phosphorylation (OXPHOS) were up-regulated, hence indicating that sZJD-28 might redirect more metabolic flux from fermentation to respiration compared with CEN.PK113-11C. These transcriptional changes also correlated well with the observed difference in growth rates between the two strains as the lower ATP generation rate through respiration instead of glycolysis resulted in a lower growth rate [15]. Besides, sZJD-28 showed increased transcriptional levels of many other metabolic pathways, such as mevalonate pathway, shikimate pathway, fatty acid biosynthesis and *β*-oxidation pathway. Altogether, these results indicated that Crabtree negative sZJD-28 redistributes the carbon and electron fluxes to strengthen carbon metabolism. These transcriptional changes in Crabtree negative sZJD-28 may further imply an elevated biosynthesis capacity compared with Crabtree positive CEN.PK113-11C when growing in the presence of excess glucose.

### Evaluating the capacity of Crabtree negative *S. cerevisiae* to produce non-ethanol chemicals

The transcriptional levels of genes related to OXPHOS were significantly improved in sZJD-28 compared with CEN.PK113-11C (Fig. 1b), indicating that the cytoplasmic NAD^+^ regeneration in Crabtree negative *S. cerevisiae* may be restrained due to the abolished ethanol generation. Thus, an NADH-consuming pathway might relieve the pressure of redox imbalance in sZJD-28.

On the other hand, capacity of biosynthetic pathways with many genes up-regulated might be increased as well. For example, up-regulations of transcriptional levels were generally occurred in PPP, shikimate pathway, MVA pathway and fatty acids pathways. Although some genes in PPP and shikimate pathway were down-regulated, such as *ZWF1* and *ARO7*, the numbers of up-regulated genes were clearly larger than the down-regulated ones. In addition, it is different to confirm the capacity of whole pathway by specific gene’ transcriptional level. This necessitates the demonstration of capacities for different pathways with many up-regulated genes. To confirm the possible enhanced biosynthetic capacity of sZJD-28, different pathways were constructed, based on the transcriptome analysis of both CEN.PK113-11C and sZJD-28, to detect generation of products from four representative metabolic nodes.

#### Biosynthesis of 2,3-butanediol and lactate from pyruvate

**Fig. 2:**
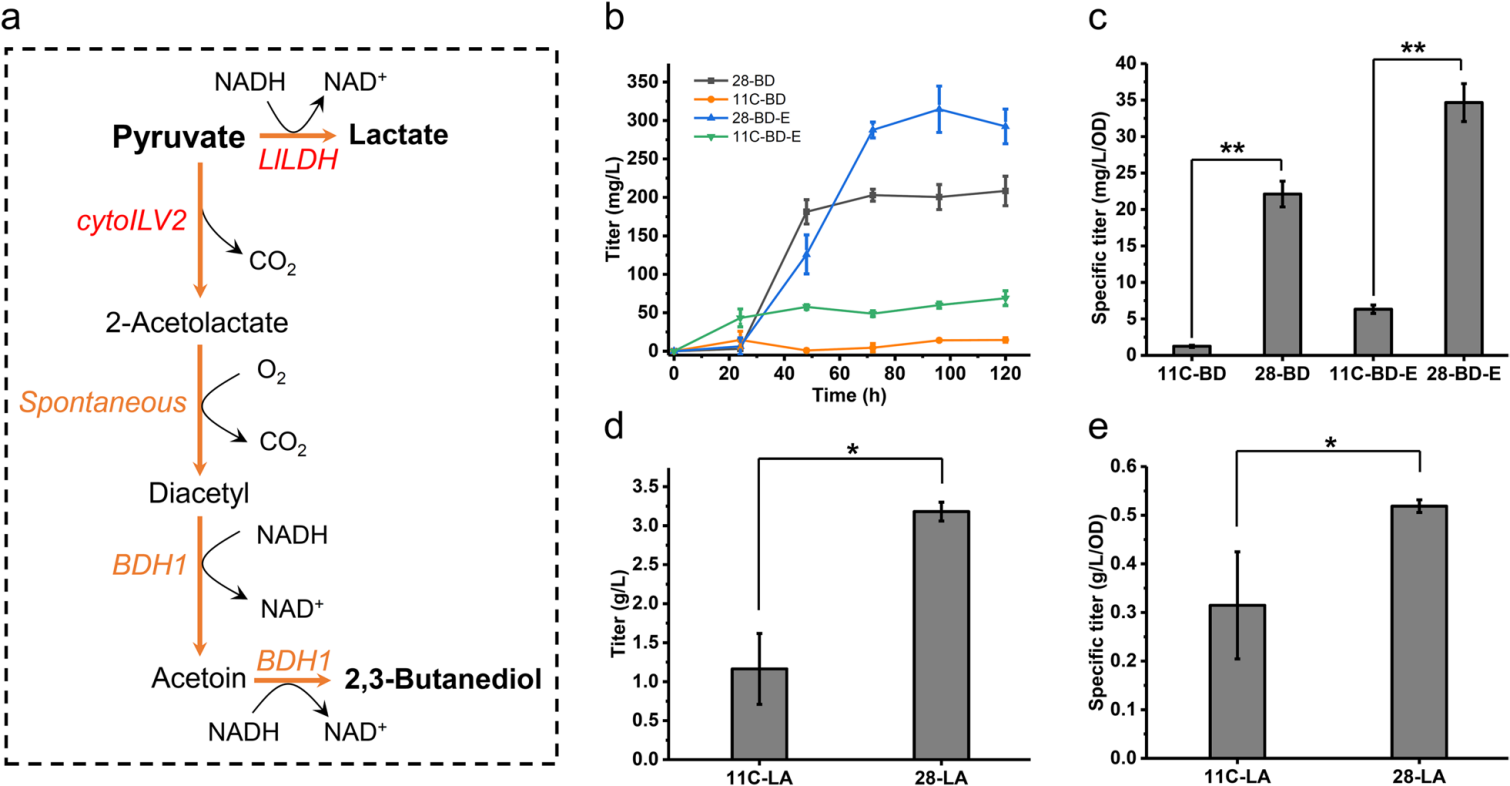
Production of 2,3-BD and lactate by Crabtree positive and negative *S. cerevisiae*. **a** Schematic representation of 2,3-BD and lactate biosynthetic pathways in *S. cerevisiae.* Both lactate dehydrogenase (*LlLDH*) from *Lactococcus lactis* and cytoplasm-relocated acetolactate synthase (*cytoILV2*) are shown in red, and yeast native steps, including spontaneous reaction and NAD-dependent (*R*,*R*)-butanediol dehydrogenase (*BDH1*) are shown in orange. **b** Titer of 2,3-BD produced by Crabtree positive 11C-BD and Crabtree negative 28-BD; **c** specific titer of 2,3-BD in Crabtree positive 11C-BD and Crabtree negative 28-BD; **d** titer of lactate produced by Crabtree positive 11C-LA and Crabtree negative 28-LA; **e** specific titer of lactate in Crabtree positive 11C-LA and Crabtree negative 28-LA. **p value < 0.005, *p value < 0.05

The production of pyruvate-derived, NADH-dependent 2,3-butanediol (2,3-BD) and lactate were first compared in sZJD-28 and CEN.PK113-11C. *S. cerevisiae* possesses an inherent pathway for generating 2,3-BD from pyruvate (Fig. 2a). As previously reported, an overexpression of acetolactate synthase (*ILV2*) is sufficient for the production of detectable level of 2,3-BD in *S. cerevisiae* [16]. Therefore, plasmid-based *ILV2* expression was adapted to test the capacity of sZJD-28 and CEN.PK113-11C to produce this compound. It was observed that strain 28-BD, harboring empty plasmid, already produced 208.4 mg/L of 2,3-BD, which was higher than the titer of strain 11C-BD by more than 13 folds, while the specific titer (g/L/OD) was improved by 16.8 folds (Fig. 2b). The yield of 28-BD was also more than 13 folds higher than that of 11C-BD as there was no residual glucose. In addition, the titer of 2,3-BD in *ILV2* overexpression strain 28-BD-E was increased to 292.3 mg/L, higher than that of strain 11C-BD-E by over threefolds (Fig. 2b), while the specific titer of 2,3-BD for 28-BD-E was 4.5 folds higher compared with 11C-BD-E (Fig. 2c). These results indicated that the imbalanced NADH level may drive the production of NADH dependent chemicals, such as 2,3-BD, although the transcriptional level of *ILV2* was similar between sZJD-28 and CEN.PK113-11C (Additional file 1: Fig. S2). Compared with Crabtree positive strain with different background, such as BY4742, 28-BD also shows fourfold higher yield of 2,3-BD (0.010 vs 0.002 g/g glucose) [17].

Lactate is another NADH-dependent chemical derived from pyruvate (Fig. 2a). However, *S. cerevisiae* lacks a native lactate dehydrogenase (*LDH*) for lactate production, but it contains a mitochondrial LDHs (*CYB2* and *DLD1*) that convert lactate to pyruvate. To test the potential advantage on cytosolic NADH provision of sZJD-28, an NADH-dependent LDH from *Lactococcus lactis* was introduced into CEN.PK113-11C and sZJD-28, resulting in strains 11C-LA and 28-LA, respectively. The results showed that 3.18 g/L of lactate was accumulated by 28-LA, while strain 11C-LA produced lactate with a peak value at 1.16 g/L. However, the latter consumed the lactate when glucose was exhausted (Fig. 2d, Additional file 1: Fig. S3). At the same time, the maximal specific titer of 28-LA was 64.9% higher than that of 11C-LA (Fig. 2e).

In *S. cerevisiae*, *CYB2* and *DLD1* are two genes involved in lactate utilization, whose expression are repressed by glucose and derepressed in ethanol or lactate [18, 19]. In sZJD-28, the transcriptional level of *CYB2* was increased by 1.3-fold, while the transcriptional level of *DLD1* was only decreased by 17% (Additional file 1: Fig. S2). With the significant decreased transcriptional levels of low-affinity glucose transporters *HXT1* and *HXT3* compared with CEN.PK113-11C (Additional file 1: Fig. S2), glucose repression could have been partially relieved in sZJD-28, resulting in the improved transcriptional level of *CYB2*. Strain 28-LA accumulated lactate instead of consuming it as it was the case for 11C-LA, demonstrating the dominant role of NADH consumption in Crabtree negative strain sZJD-28. Overall, the above results indicated that sZJD-28 possessed more efficient pyruvate and NADH supply compared with CEN.PK113-11C. In addition, lactate production of Crabtree positive strain CEN.PK2-1C harboring different *LDH*s reached a maximal titer of 2.50 g/L [20], lower than that of 28-LA (3.18 g/L).

#### *p*-Coumaric acid biosynthesis from shikimate pathway

The shikimate pathway is usually applied in the synthesis of aromatic amino acids (AAAs) and their derivatives (AAADs) which serve as precursors for various industrially relevant compounds [21]. For overproduction of shikimate derivatives in *S. cerevisiae*, enhancing the expression of native genes such as *ARO1-4* and *ARO7-9* is the common metabolic engineering strategy [22–27]. As shown in Fig. 1c, increased transcriptional level of phenylalanine and tyrosine biosynthesis such as the *ARO* genes and their precursor biosynthesis pathway PPP were observed for sZJD-28 compared with CEN.PK113-11C. Furthermore, Pdc5p consume precursors of AAAs, para-hydroxy-phenylpyruvate and phenylpyruvate into fusel alcohol/acid [28]. The deficiency of Pdc5p sZJD-28 may be advantageous for the biosynthesis of AAAs and AAADs. These results indicated that sZJD-28 could be a superior strain for synthesizing aromatic chemicals compared with CEN.PK113-11C.

**Fig. 3:**
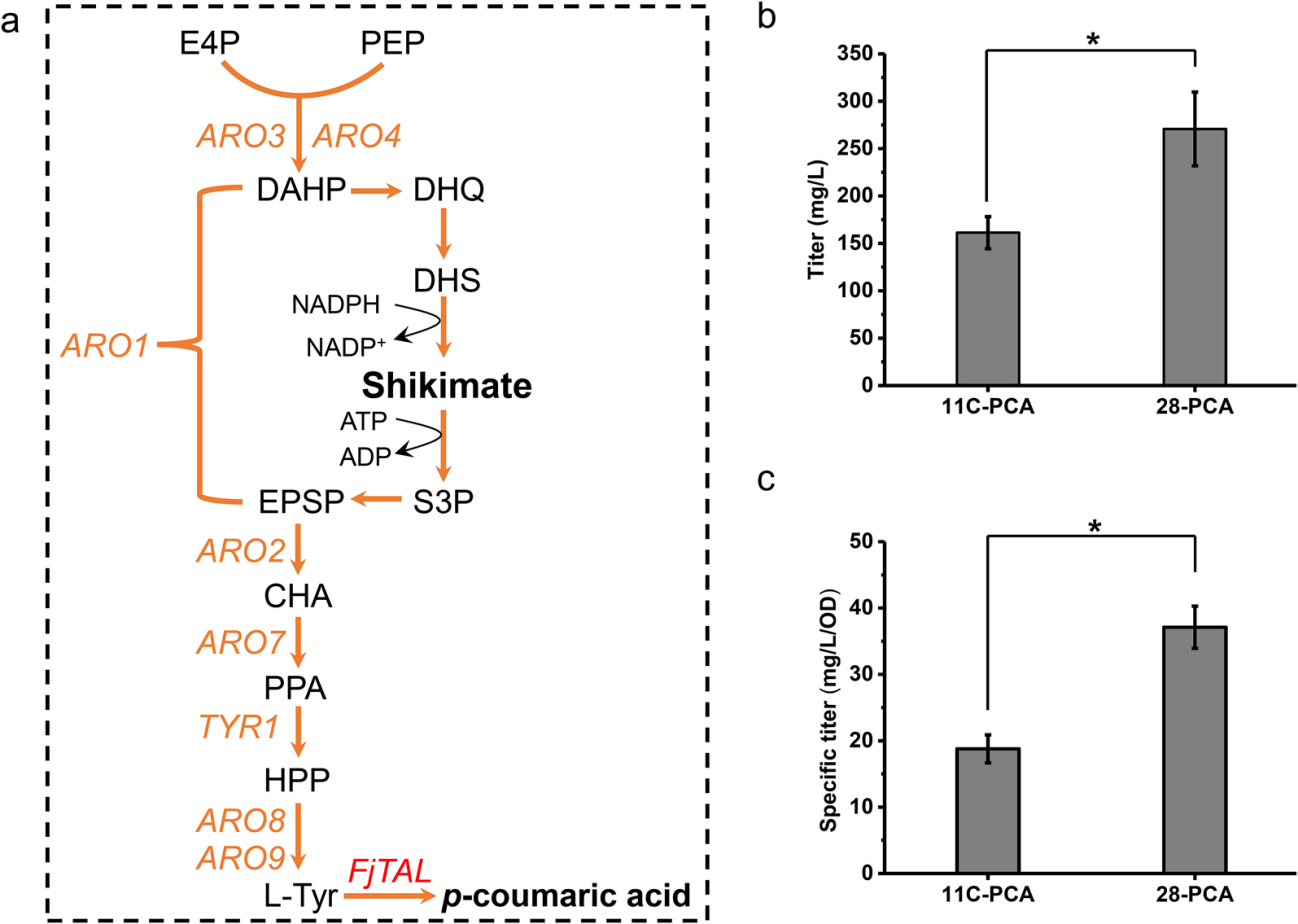
Production of *p*-coumaric acid by Crabtree positive and negative *S. cerevisiae*. **a** Schematic representation of *p*-coumaric acid biosynthetic pathways in *S. cerevisiae*. Yeast native genes, including pentafunctional arom protein (*ARO1*), bifunctional chorismate synthase/riboflavin reductase (*ARO2*), 3-deoxy-7-phosphoheptulonate synthase (*ARO3*, *ARO4*), chorismate mutase (*ARO7*), aromatic aminotransferase I (*ARO8*), aromatic aminotransferase II (*ARO9*), and prephenate dehydrogenase (*TYR1*) are shown in orange. Tyrosine ammonia lyase (*FjTAL*) from *Flavobacterium johnsoniae* is shown in red. **b** Titer of *p*-coumaric acid produced by Crabtree positive 11C-PCA and Crabtree negative 28-PCA; **c** specific titer of* p*-coumaric acid in Crabtree positive 11C-PCA and Crabtree negative 28-PCA. *p value < 0.05

To confirm the above hypothesis, *p*-coumaric acid was selected as a representative AAAD for subsequent experiments. The tyrosine ammonia lyase (*FjTAL*) from *Flavobacterium johnsoniae*, which could covert tyrosine into *p*-coumaric acid, was expressed in sZJD-28 and CEN.PK113-11C resulting in 28-PCA and 11C-PCA, respectively (Fig. 3a). The production of *p-*coumaric acid by 28-PCA reached 270.7 mg/L, which was 67.8% higher than that of 11C-PCA (Fig. 3b), and the yield was also 67.8% higher than that of 11C-PCA due to no detected residual glucose. The specific titer in 28-PCA was increased by 97.5% than that of 11C-PCA (Fig. 3c). These results proved that the Crabtree negative *S. cerevisiae* sZJD-28 could display enhanced capacity for AAADs biosynthesis compared with the Crabtree positive *S. cerevisiae* CEN.PK113-11C. For other Crabtree positive *S. cerevisiae*, CEN.PK102.5B and BY4741-based *p*-coumaric acid producers reached titers of 0.202 and 0.081 g/L [29], which were lower than that of 28-PCA (Fig. 3b).

#### Farnesene and lycopene biosynthesis from acetoacetyl-CoA

**Fig. 4:**
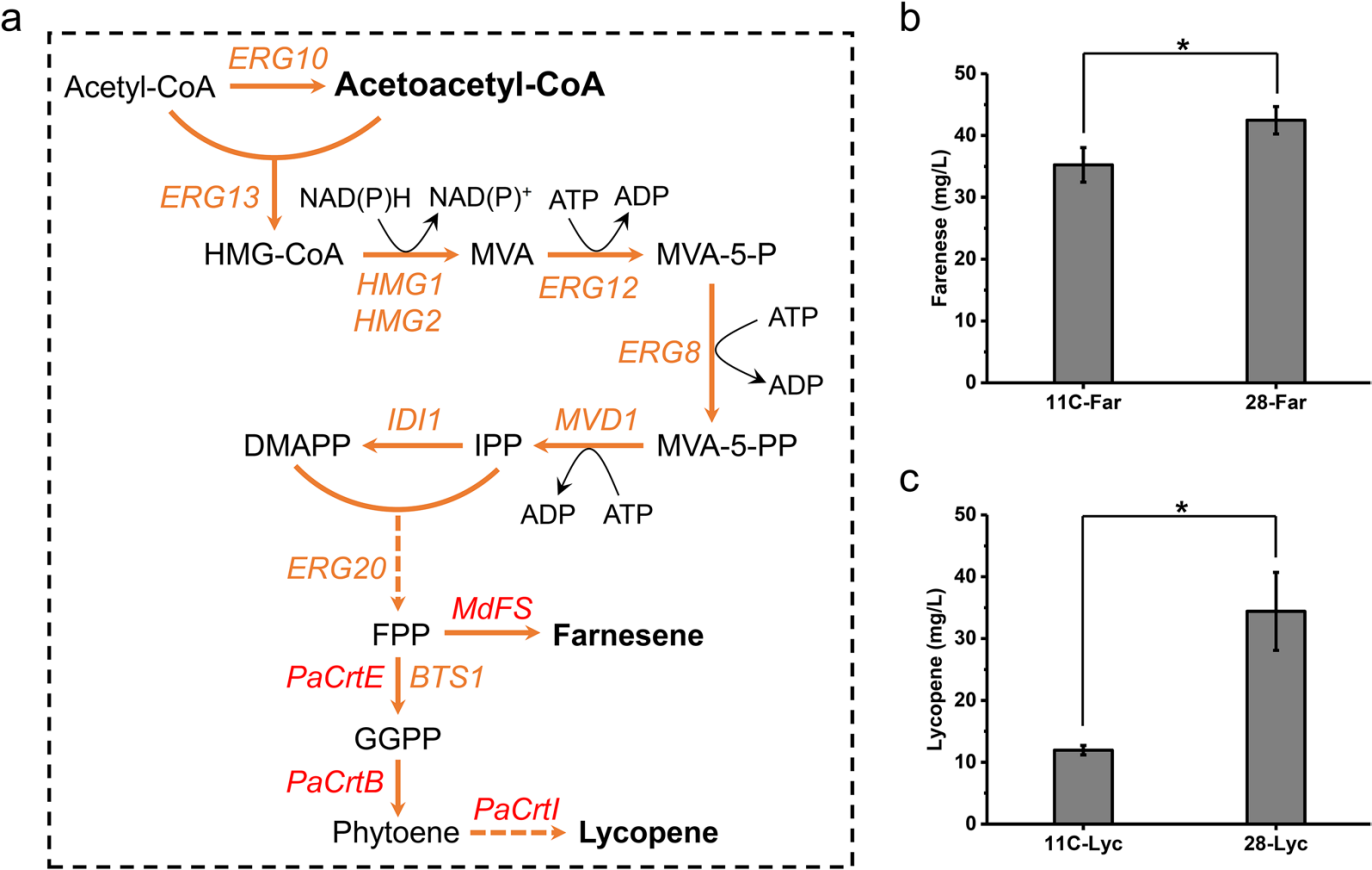
Production of farnesene and lycopene by Crabtree positive and negative *S. cerevisiae*. **a** Schematic representation of farenese and lycopene biosynthetic pathways in *S. cerevisiae*. Yeast native genes including phosphomevalonate kinase (*ERG*8), acetyl-CoA C-acetyltransferase (*ERG10*), mevalonate kinase (*ERG12*), 3-hydroxy-3-methylglutaryl-CoA (HMG-CoA) synthase (*ERG13*), farnesyl pyrophosphate synthetase (*ERG20*), HMG-CoA reductase (*HMG1*, *HMG2*), isopentenyl diphosphate: dimethylallyl diphosphate isomerase (*IDI1*), mevalonate pyrophosphate decarboxylase (*MVD1*), and geranylgeranyl diphosphate synthase (*BTS1*) are shown in orange. Farnesene synthase (*MdFS*) from *Malus domestica*, geranylgeranyl pyrophosphate synthase (*PaCrtE*) from *Pantoea ananatis*, phytoene synthase (*PaCrtB*) from *Pantoea ananatis*, and phytoene desaturase (*PaCrtI*) from *Pantoea ananatis* are shown in red. **b** Titer of farnesene produced by Crabtree positive 11C-Far and 28-Far. **c** Titer of lycopene produced by Crabtree positive 11C-Lyc and Crabtree negative 28-Lyc. *p value < 0.05

The MVA pathway is usually used by *S. cerevisiae* to synthesize isoprenoids [30, 31]. In sZJD-28, the transcriptional level of this pathway was clearly higher than that of CEN.PK113-11C (Fig. 1c). Acetyl-CoA C-acetyltransferase (Erg10p) serves as the first enzyme in the MVA pathway [32], whereas HMG-CoA reductase (Hmg2p) [33] and isopentenyl pyrophosphate isomerase (Idi1p) [34] play rate-limiting roles in sterol biosynthesis. Mevalonate pyrophosphate decarboxylase (Mvd1p) [35], farnesyl pyrophosphate synthetase (Erg20p) [36] and geranylgeranyl diphosphate synthase (Bts1p) [37] serve as essential enzymes involved in the biosynthesis of isoprenoids (Fig. 4a). Increasing the transcription levels of these genes in *S. cerevisiae* have already been proven to improve the production of isoprenoids [38–42].

To demonstrate the potential strength of sZJD-28 on isoprenoids biosynthesis, two representative isoprenoids, namely, farnesene and lycopene, were selected. Overexpression of farnesene synthase rendered farnesene production in both sZJD-28 and CEN.PK113-11C, resulting in strains 28-Far and 11C-Far. The titer of farnesene in strain 28-Far was 20.5% higher than that of strain 11-Far (Fig. 4b). Crabtree positive strain YPH499 with farnesene synthase produced farnesene in titer of approximate 12.5 mg/L [43], lower than that of 28-Far (Fig. 4b). Similarly, after introducing *CrtE*, *CrtB* and *CrtI*, the three genes encoding key enzymes for lycopene biosynthesis, into the sZJD-28 and CEN.PK113-11C, the lycopene production of strain 28-Lyc reached 34.4 mg/L which was 1.88 times greater than that of strain 11-Lyc (Fig. 4c). The improvements in the yields of farnesene and lycopene are the same as the final titers as there was not detected any residual glucose. Overall, the biosynthetic capacity of isoprenoids with acetoacetyl-CoA as precursor was stronger in Crabtree negative sZJD-28 compared with Crabtree negative CEN.PK113-11C. Specific titer of 28-Lyc reached 13.51 mg/g DCW, which was much higher than those of lycopene-producers derived from Crabtree positive strains, such as CEN.PK2-1D (approximate 5 mg/g DCW) [44] and BY4741 (approximate 4 mg/g DCW) [45].

#### 3-Hydroxypropionic acid and free fatty acids biosynthesis from malonyl-CoA

**Fig. 5:**
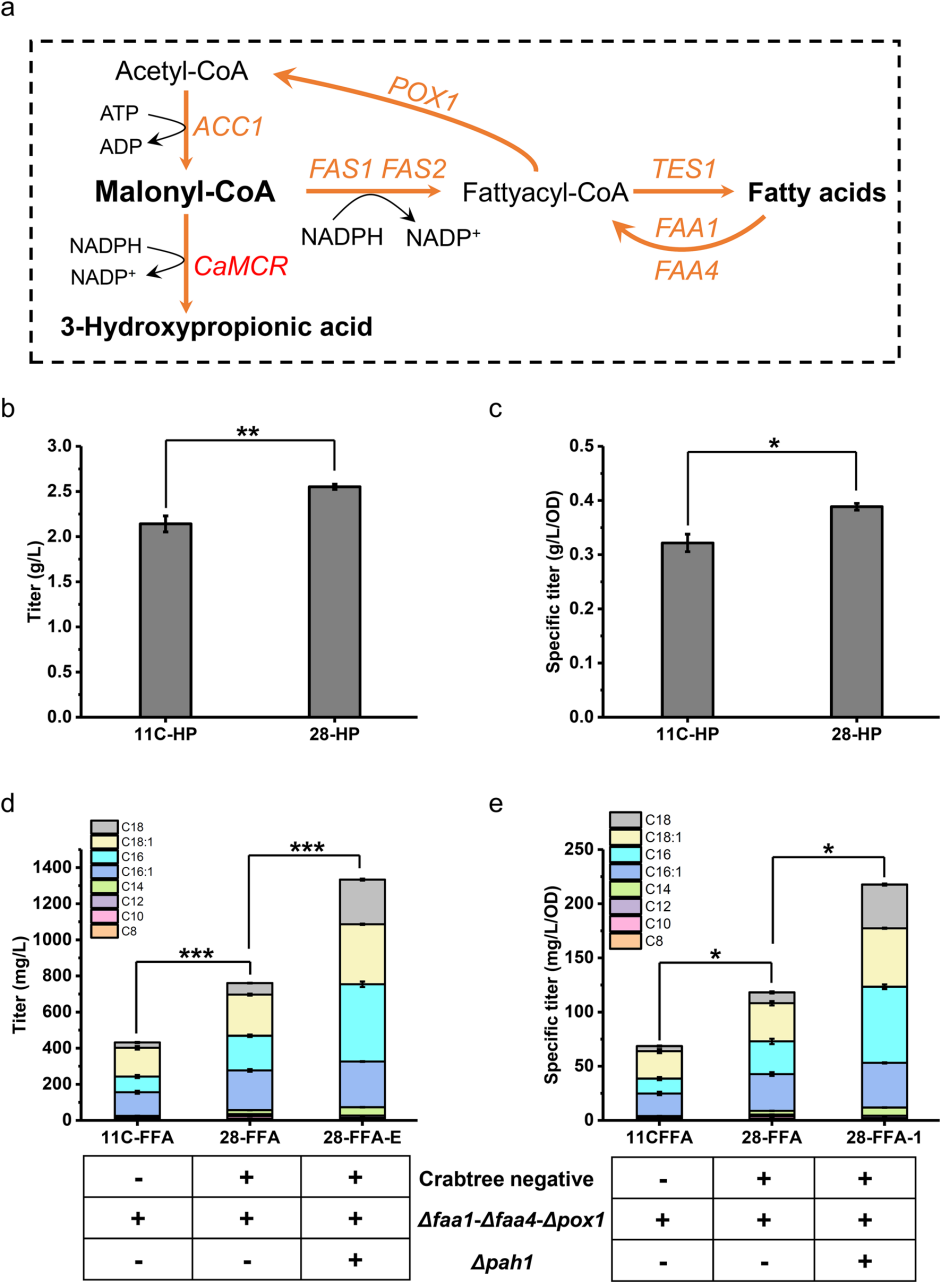
Production of 3-HP and free fatty acids by Crabtree positive and negative *S. cerevisiae*. **a** Schematic representation of 3-HP and free fatty acids biosynthetic pathway in *S. cerevisiae*. Yeast native genes, including acetyl-CoA carboxylase (*ACC1*), beta subunit of fatty acid synthetase (*FAS1*), alpha subunit of fatty acid synthetase (*FAS2*), peroxisomal acyl-CoA thioesterase (*TES1*), long chain fatty acyl-CoA synthetase (*FAA1*, *FAA4*), and fatty-acyl coenzyme A oxidase (*POX*1) are shown in orange. Malonyl-CoA reductase (*CaMCR*) from *Chloroflexus aurantiacus* is shown in red. **b** Titer of 3-HP produced by Crabtree positive 11C-HP and negative 28-HP; **c** Specific titer of 3-HP in Crabtree positive 11C-HP and negative 28-HP; **c** titer of FFA produced by Crabtree positive 11C-FFA, negative 28-FFA and 28-FFA-E; **d** specific titer of FFA in Crabtree positive 11C-FFA, negative 28-FFA and 28-FFA-E. ***p value < 0.001; **p value < 0.005; *p value < 0.05

In addition to the above metabolic nodes, malonyl-CoA is also an important precursor for chemical biosynthesis (Fig. 5a). In this case, the transcriptional analysis indicated that malonyl-CoA derived fatty acid metabolism in sZJD-28 was more active compared with CEN.PK113-11C (Fig. 1c). In particular, the transcriptional level of *ACC1*, involved in the generation of malonyl-CoA in sZJD-28, was 74.8% higher than in CEN.PK113-11C (Additional file 1: Fig. S4).

To confirm these results, 3-hydroxypropionic acid and free fatty acids, the malonyl-CoA derivatives, were selected to determine the potential advantage of sZJD-28. The resulting strains 11C-HP and 28-HP were obtained after heterologous expression of malonyl-CoA reductase (*MCR1*) from *Chloroflexus aurantiacus* in CEN.PK113-11C and sZJD-28. As expected, 3-HP production in 28-HP was 19.2% higher than in 11C-HP (Fig. 5b), and the specific titer was 20.8% higher (Fig. 5c). In addition, compared with a reported study about the production the 3-HP by Crabtree positive strains, sZJD-28 also showed a 4.57-fold higher titer [46], also indicating the higher biosynthetic capacity of Crabtree negative strains. As previously reported, simultaneously deleting *FAA1*, *FAA4* and *POX1* enabled *S. cerevisiae* to accumulate free fatty acids (FFA) [47]. Therefore, these three genes were deleted in sZJD-28 and CEN.PK113-11C, resulting in strains 28-FFA and 11C-FFA. Compared with 11C-FFA, 28-FFA showed higher production of free fatty acids by 76.3%, reaching 780.4 mg/L (Fig. 5c) and higher specific titer by 71.5% (Fig. 5d). The improvements in yields of 3-HP and FAA also reached 19.2% and 76.3% as no residual glucose. Besides the key gene *ACC1*, the up-regulated *FAS1* and *FAS2* (Additional file 1: Fig. S2) could also have contributed to the improved capacity for fatty acids biosynthesis of sZJD-28.

Compared with Crabtree positive strains, Crabtree negative ones showed superior biosynthetic capabilities for chemicals derived from multiple metabolic nodes. Of these, lactate and 2,3-BD derived from pyruvate were significantly improved, while *p*-coumaric acid derived from shikimate, 3-HP derived from malonyl-CoA, and farnesene derived from acetoacetyl-CoA showed modest improvements in the Crabtree negative strain. This was probably due to needs for different cofactors for different pathways. Ethanol production consumes the surplus NADH generated by glycolysis, such that deleting ethanol synthesis leads to accumulation of NADH in the Crabtree negative strain. The biosynthesis of 2,3-BD and lactate requires the use of the cofactor NADH and this ensures a balance of NADH production in the cytosol during the conversion of glucose to pyruvate [48, 49]. In the shikimate pathway, NAD^+^ instead of NADH is needed for Pha2p and Try1p, and hence, a similar balance in NADH is not achieved when using this pathway. *MCR1* from *Chloroflexus aurantiacus* [50] used for the production of 3-HP and over-expression of *HMGR* [51] in the MVA pathway are both NADPH-dependent. Consequently, for these products there was no balancing of NADH. The different cofactors needed for producing the different products may, therefore, result in varying performance during improving biosynthesis by the Crabtree negative chassis strain.

### Fed-batch fermentation of Crabtree negative FFA hyperproducing *S. cerevisiae*

**Fig. 6:**
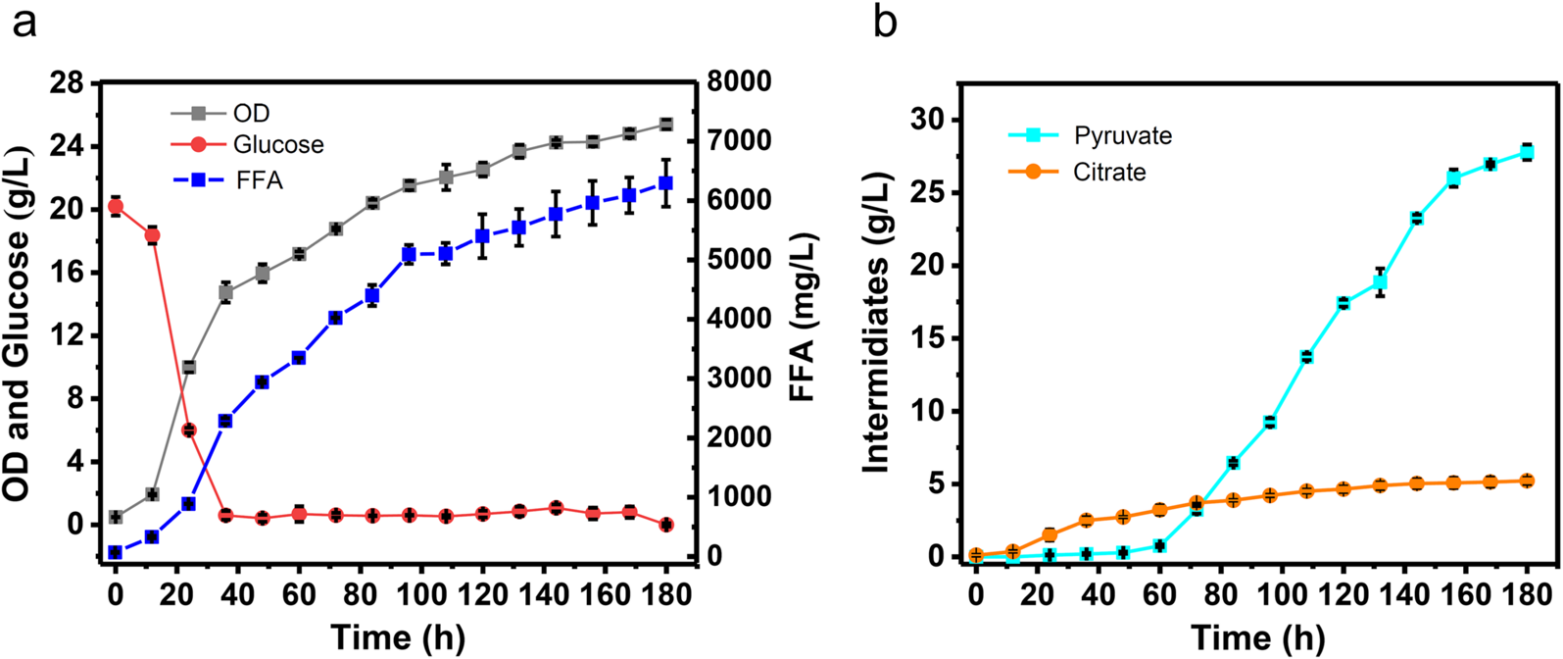
Fed-batch fermentation of FFA in 28-FFA-E. Cultivation was carried out in minimal salt medium with pH 5.0 adjusted by KOH. The dissolved oxygen concentration was maintained above 20% saturation by automatic adjustment of the stirring rate. Glucose feeding was initiated at 40 h. **a** FFA production, OD and glucose consumption by Crabtree negative 28-FFA-E. FFA titer is the sum of all detected FFA, including C8, C10, C12, C14, C16:1, C16, C18, C18:1; **b** production of pyruvate and citrate during fed-batch fermentation of FFA in Crabtree negative 28-FFA-E

As reported, deleting *PAH1* could stimulate FFA production in the Crabtree positive *S. cerevisiae* due to the up-regulation of fatty acid biosynthesis indirectly by phosphatidic acid [47]*.* To construct a Crabtree negative FFA hyperproducer, *PAH1* was, therefore, deleted based on strain 28-FFA. The resulting strain 28-FFA-E showed 75.3% and 86.7% higher FFA titer and specific titer, respectively, compared with 28-FFA. reaching 1333.2 mg/L and 217.7 mg/L/OD (Fig. 5e). To investigate the biosynthetic capacity of this FFA producer, fed-batch fermentation of 28-FFA-E was conducted in a 5 L bioreactor. During the fermentation process, glucose consumption, cell growth as well as production of FFA and intermediates were monitored. Glucose feeding was initiated at 40 h and glucose concentration was kept below 1 g/L. KOH was used to adjust pH to 5.0 and the dissolved oxygen was maintained above 20% by automatic adjustment of stirring rate. After 180 h, the titer of FFA reached 6295.6 mg/L (Fig. 6a).

Although fed-batch production of FFA was lower than the highest titer reported for a Crabtree positive *S. cerevisiae* strain, i.e., Y&Z036 (33.4 g/L) [52], the specific titer of 28-FFA-E reached 247.7 mg/L/OD which is over 2 folds higher than that of Y&Z036. In addition, the FFA content of 28-FFA-E reached 897.3 mg/g DCW (according to 3.55 OD = 1 g DCW/L [53]), and as such it was comparable with the content of oleaginous yeast *Yarrowia lipolytica* (1200 mg/g DCW) [54] and higher than the specific titer of *E. coli* (about 470 mg/g DCW) [55] according to 1 OD = 0.379 g DCW/L [56]. It is worth noting that specific titers reached close to and higher than the 1000 mg/g DCW due to the secretion of fatty acids into the culture broth. Despite of the lower FFA titer of 28-FFA-E than the Crabtree positive *S. cerevisiae* strain Y&Z036 [52], 28-FFA-E still showed great potential in FFA biosynthesis, because there was much less genetic optimization performed in 28-FFA-E. Furthermore, as shown in Fig. 6b, the high accumulation of pyruvate and citrate in the fed-batch fermentation process, reached 27.8 g/L and 5.2 g/L, respectively, thereby causing carbon loss and low biomass. To overcome these problems, increasing the copy of PO-PTA and heterogenous expression of ATP-citrate lyase, converting pyruvate and citrate to acetyl-CoA, respectively, could relieve intermediates accumulations, thus resulting in more carbon towards the biosynthesis of targeted chemicals.

## Conclusions

In this study, comparative transcriptome analysis between Crabtree positive and the fast-growing Crabtree negative *S. cerevisiae* was demonstrated for the first time. It turned out that transcriptional levels of genes related to OXPHOS and central metabolic pathways were changed significantly. This analysis provided key information about the potential pathways for non-ethanol chemicals production, which is helpful to choose chemicals synthesized in this superior chassis cell. The following demonstration of productions from various metabolic nodes confirmed the advantages of Crabtree negative strains in yield, titer and specific titer. In addition, this study collectively showed the preferable feature of Crabtree negative strains in NADH-consuming chemicals biosynthesis by balancing redox homeostasis. Among these chemicals, compared with Crabtree positive strain, productions of NADH dependent chemicals 2,3-BD and lactate in Crabtree negative strains were significantly improved by 16.8 and 1.65 folds in titers, respectively; Productions of *p*-coumaric acid, farnesene, lycopene, 3-HP and FFA from corresponding pathways with up-regulated genes in Crabtree negative strains were improved by 0.19–1.88-folds in titer; In fed-batch, Crabtree negative 28-FFA-E reached a highest reported specific titer of FFA in *S. cerevisiae*. This research not only deepened the current understanding of the carbon metabolism of Crabtree negative *S. cerevisiae*, but also proved that such a strain possesses inherent superiority for chemicals biosynthesis, thereby providing an alternative chassis cell for synthetic biology.

## Methods

### Culture conditions and media

*Escherichia coli* DH5a was routinely used for plasmid propagation in LB medium, consisting of 10 g/L tryptone, 5 g/L yeast extract and 10 g/L NaCl.

*Saccharomyces cerevisiae* harboring different biosynthetic pathways was transferred from glycerol stock to 5 mL minimal medium, consisting of the following components: 20 g/L glucose, 14.4 g/L KH_2_PO_4_, 7.5 g/L (NH_4_)_2_SO_4_, 0.5 g/L MgSO_4_·7H_2_O, trace metal, vitamin solution, 40 mg/L histidine and/or 40 mg/L uracil according to auxotroph [57]. Transfer the culture to 20 mL same medium with initial OD of 0.05 when strains were grown to *log-phase*, and fermentation broth were sampled for analysis at 120 h. For CEN.PK113-11C and sZJD-28 derived strains, samples were taken between 0 and 120 h, which is longer enough to consume carbon source and suitable for evaluate the biosynthetic capacity of chemicals.

### Transcriptome analysis

Glycerol stocks of sZJD-28 and CEN.PK113-11C were transferred into 5 mL YPD (20 g/L glucose, 20 g/L peptone and 10 g/L yeast extract) medium, and incubated at 30 °C, 250 rpm for 24 h. Cells were harvested by centrifuging at 12,000 rpm for 1 min. Supernatants were discarded and cells were washed by sterile water for twice. Cells were resuspended in 20 mL minimal medium with initial OD of 0.05, pH 6.0, consisting of the following components: 20 g/L glucose, 14.4 g/L KH_2_PO_4_, 7.5 g/L (NH_4_)_2_SO_4_, 0.5 g/L MgSO_4_·7H_2_O, trace metal, vitamin solution, 40 mg/L histidine and 40 mg/L uracil [57], and incubated at 30 °C, 250 rpm. Cells were sampled for transcriptome analysis when OD600 reached 1.

After sampling, total RNA was extracted using the Yeast RNA Kit (Omega). The RNA sequencing was carried out in Novogene Co., Ltd. (Beijing, China). Illumina RNA-Seq data were aligned on the yeast genome using Bowtie2 [58]. Transcript abundance was estimated by method RSEM [59]. Differential expression analysis was processed by edgeR package in the R programming language [60]. Reporter analysis on GO terms and transcription factors was carried out via the Platform for Integrative Analysis of Omics (PIANO) R package [13]. GO slim mapping file was downloaded from SGD (http://sgd-archive.yeastgenome.org/) and gene-TF interaction pairs were obtained from YeasTMine (http://yeastmine.yeastgenome.org).

### Construction of biosynthesis pathways

L-lactate dehydrogenase (*LlLDH*) was from *Lactococcus lactis* NZ9000. Acetolactate synthase (*cytoILV2*) was native *S. cerevisiae* gene but was truncated (2–90 aa) [8] to relocate it to the cytoplasm. Both *LlLDH* and *cytoILV2* were inserted into plasmid pSP-GM2 between the restriction sites BamHI and NheI to construct plasmids used for production of lactate and 2,3-BD, respectively. Malonyl-CoA reductase (*CaMCR*), α-farnesene synthase (*MdFS*) and tyrosine ammonia lyase (*FjTAL*), from *Chloroflexus aurantiacus* [46], *Malus domestica* [61] and *Flavobacterium johnsoniae* [62], respectively, were codon optimized and placed under the control of *TEF1* promoter, with their expression cassettes subsequently integrated into chromosomal site *XII-2* to construct strains producing 3-HP, farnesene and *p*-coumaric acid. Similarly, geranylgeranyl pyrophosphate synthase (*PaCrtE*), phytoene synthase (*PaCrtB*) and phytoene desaturase (*PaCrtI*), all from *Pantoea ananatis*, were codon optimized, with their expression placed under the control of the promoters *CDC19*, *CCW12* and *TDH3,* respectively. As before, the expression cassettes were then integrated into chromosomal site *XII-2* to construct lycopene-producing strains. FFAs-producing strains were constructed by deleting *FAA1*, *FAA4*, *POX1* and *PAH1*.

Expression formats were chosen according to previous study. As reported, 2,3-BD [16, 63–65] and lactate [20, 66–68] were widely produced by expressing genes in plasmids; meanwhile, *p*-coumaric acid [21, 29, 69–71], lycopene [44, 45, 72, 73], farnesene [3, 43, 74] and 3-HP [75] were widely produced by integrating genes into genome. Using the same expression format as reported will be of referential significance for demonstrating the biosynthetic capacity of chassis cell.

All the plasmids and expression cassettes were transformed into yeast by LiAC/ssDNA method [76]. Expression cassettes were integrated by CRISPR/Cas9 system. All strains used in this study are listed in Table 1. All primers, gRNA and deleting donors used in this study were summarized in Additional file 1: Tables S1 and S2.

### Metabolite extraction and analysis

3-HP and lactate were measured at 65 °C by an HPLC system equipped with a refractive-index detector and a Bio-Rad HPX-87H column using 0.5 mM H_2_SO_4_ as mobile phase at a flow rate of 0.6 mL min^−1^. Esterification and analysis of FFAs were performed as previously described [78]. 2,3-BD was detected by the same methods as 3-HP, except for the use of 5 mM H_2_SO_4_ as the mobile phase and column temperature of 28 °C. Lycopene and farnesene were detected by HPLC equipped with an Innoval C18 column. The extracted and analyzed method was described previously [45]. *p*-Coumaric acid was detected by HPLC equipped with Discovery HS F5 150 mm × 2.1 mm column, as described in a previous study [62].

All data were presented as the mean of three biological replicates with error bars representing the standard deviations. *P* values were generated by performing* t* tests for determining statistical significance.

### Fed-batch fermentation of free fatty acids in 5 L bioreactor

*Saccharomyces cerevisiae* was transferred from glycerol stock to 5 mL SD-URA medium and incubated at 30 °C and 250 rpm. Transfer the culture to 200 mL minimal medium with initial OD of 0.05. Once the strains reached the *log-phase*, they were transferred to a 5 L bioreactor containing 1.5 L batch medium, pH 6.0, consisting of 20 g/L glucose, 5 g/L (NH_4_)_2_SO_4_, 3 g/L KH_2_PO_4_, 0.5 g/L MgSO_4_·7H_2_O, 40 mg/L URA, 40 mg/L histidine, trace metal and vitamin solutions. Fed batch medium contained 600 g/L glucose, and 7 g/L (NH_4_)_2_SO_4_, 15 g/L KH_2_PO_4_, 2.5 g/L MgSO_4_·7H_2_O, 200 mg/L URA, 200 mg/L histidine, fivefolds of trace metal and vitamin solutions.

## Supplementary Information

**Additional file 1: Figure S1.** Reporter transcription factors (TFs) analysis of strain sZJD-28 compared with CEN.PK 113-11C. **Figure S2.** Fold changes of transcriptional levels of *ILV2*, *HXT1*, *HXT3*, *CYB2*, *DLD1*, *ACC1*, *FAS1* and *FAS2* in sZJD-28 relative to CEN.PK113-11C. **Figure S3.** Production of lactate by Crabtree positive 11C-LA and negative 28-LA. **Table S1.** Primers used in this study. **Table S2.** gRNA and deleting donors used in this study.

## Data Availability

The data sets generated during this study are included in this article and its additional files.
