# Supplementary material for: A highly efficient transcriptome-based biosynthesis of non-ethanol chemicals in Crabtree negative Saccharomyces cerevisiae
Source: Biotechnol Biofuels Bioprod. 2023 Mar 4;16:37. doi: 10.1186/s13068-023-02276-5 (PMC9985264; doi:10.1186/s13068-023-02276-5)
Supplement: Supplementary file 1 — Additional file 1: Figure S1. Reporter transcription factors (TFs) analysis of strain sZJD-28 compared with CEN.PK 113-11C. Figure S2. Fold changes of transcriptional levels of ILV2, HXT1, HXT3, CYB2, DLD1, ACC1, FAS1 and FAS2 in sZJD-28 relative to CEN.PK113-11C. Figure S3. Production of lactate by Crabtree positive 11C-LA and negative 28-LA. Table S1. Primers used in this study. Table S2. gRNA and deleting donors used in this study. [file 13068_2023_2276_MOESM1_ESM.docx]

**Fig. S1** Reporter transcription factors (TFs) analysis of strain sZJD-28 compared with CEN.PK 113-11C. The color key shows the rank of TFs and the significance (p-value) of the TF is included in each cell of the heatmap. TFs that have a consensus rank ≤5 in any of the groups (distinct-directional down, mixed-directional down, non-directional change, mix-directional up and distinct-directional up) are shown in the heatmap. Rank of TFs was based on their *P*-value to yield a consensus score^1^.

**Fig. S2** Fold changes of transcriptional levels of *ILV2*, *HXT1*, *HXT3*, *CYB2*, *DLD1*, *ACC1*, *FAS1* and *FAS2* in sZJD-28 relative to CEN.PK 113-11C.

**Fig. S3** Production of lactate by Crabtree positive 11C-LA and negative 28-LA.

**Table S1** Primers used in this study

| Name | Sequence |
| --- | --- |
| LlLDH-S | AAGGATCCATGAAAATTAATAACAAAAAAGTTGTAATTGTTGGAGCT |
| LlLDH-A | TAGCTAGCTTACAAAGTACATTTTTCTTTAATTGAATCAAAA |
| CytoILV2-S | AAGGATCCATGGGTTTAACTGGTGGTCAAATATTTAACGA |
| CytoILV2-A | TAGCTAGCTCAGTGCTTACCGCCTGTACG |
| FjTAL-S | ATGAACACCATCAACGAATACTTGTCCTTG |
| FjTAL-A | ttaGTTGTTAATCAAGTGATCCTTAACTTTTT |
| CaMCR-S | ATGTCCGGGACAGGTCGTCTGGCCGGAAA |
| CaMCR-A | TTATACAGTTATGGCTCTCCCTCTATGA |
| MdFS-S | ATGGAATTTAGGGTTCACCTGCAAGCC |
| MdFS-A | ttaGTTTACCAAAGGTTGAAACA |
| PaCrtE-S | ATGACTGTTTGTGCAAAAAAACACG |
| PaCrtE-A | TTATGAAACTGCGGCTAACTTCTTGTC |
| PaCrtB-S | ATGAATAACCCGAGTCTGCTGAATCA |
| PaCrtB-A | TTAAAGTGGACGTTGCCACAAATGAGC |
| PaCrtI-S | ATGAATAACCCGAGTCTGCTGAATCAC |
| PaCrtI-A | TTAAatccgctctaaccgaaaaggaag |

**Table S2** gRNA and deleting donors used in this study

| Name | Sequence |
| --- | --- |
| XII-2 gRNA | TGAAACTCTAATCCTACTAT |
| POX1 gRNA | AGGGGATATTGGAGCGAAGA |
| PAH1 gRNA | TCGACACTATCAAGGCTGAC |
| FAA1 gRNA | AATGTTGTCGCAGGTGTTGG |
| FAA4 gRNA | ATGTACACTTCTGGTTCCAC |
| POX1 donor | CTAATAAGTATCACAGAAAAAAAGAAAATATAATAAATTAGTATTGCGATGTAGAGGTTT  CCTGTTTTCCTTCGAACCCTCTGTTTTGCGACTTTTGTTT |
| PAH1 donor | CTTAATATGCAGTATGGATCGTTATAAATAATATTCGGCTACAAGAATCTAATCGACCGAT  GTGTCTATCTTCAGTAATTTCTTCCCTGTATGTTTCTTA |
| FAA1 donor | GGATACAATAAAAACTAGAACAAACACAAAAGACAAAAAAAGACAACAATTGGATCAA  CATTTCCATGATAGGAAAGCCTCATCATACTAAAGCACTTTT |
| FAA4 donor | TTTTCTCTGTTCTTCACTATTTCTTGAAAAACTAAGAAGTACGCATCAAAAGGAAGACAT  AGTTTTTTACTTTCCCCCCTGCCCTTCATAAACACTACGT |

**Table S3** Sequence of pSP-GM2

| Name | Sequence | Reference |
| --- | --- | --- |
| pSP-GM2 | tcgcgcgtttcggtgatgacggtgaaaacctctgacacatgcagctcccggagacggtcacagcttgtctgtaagcggatgccgggagcagacaagcccgtcagggcgcgtcagcgggtgttggcgggtgtcggggctggcttaactatgcggcatcagagcagattgtactgagagtgcaccataccacagcttttcaattcaattcatcattttttttttattcttttttttgatttcggtttctttgaaatttttttgattcggtaatctccgaacagaaggaagaacgaaggaaggagcacagacttagattggtatatatacgcatatgtagtgttgaagaaacatgaaattgcccagtattcttaacccaactgcacagaacaaaaacctgcaggaaacgaagataaatcatgtcgaaagctacatataaggaacgtgctgctactcatcctagtcctgttgctgccaagctatttaatatcatgcacgaaaagcaaacaaacttgtgtgcttcattggatgttcgtaccaccaaggaattactggagttagttgaagcattaggtcccaaaatttgtttactaaaaacacatgtggatatcttgactgatttttccatggagggcacagttaagccgctaaaggcattatccgccaagtacaattttttactcttcgaagacagaaaatttgctgacattggtaatacagtcaaattgcagtactctgcgggtgtatacagaatagcagaatgggcagacattacgaatgcacacggtgtggtgggcccaggtattgttagcggtttgaagcaggcggcagaagaagtaacaaaggaacctagaggccttttgatgttagcagaattgtcatgcaagggctccctatctactggagaatatactaagggtactgttgacattgcgaagagcgacaaagattttgttatcggctttattgctcaaagagacatgggtggaagagatgaaggttacgattggttgattatgacacccggtgtgggtttagatgacaagggagacgcattgggtcaacagtatagaaccgtggatgatgtggtctctacaggatctgacattattattgttggaagaggactatttgcaaagggaagggatgctaaggtagagggtgaacgttacagaaaagcaggctgggaagcatatttgagaagatgcggccagcaaaactaaaaaactgtattataagtaaatgcatgtatactaaactcacaaattagagcttcaatttaattatatcagttattaccctatgcggtgtgaaataccgcacagatgcgtaaggagaaaataccgcatcaggaaattgtaaacgttaatattttgttaaaattcgcgttaaatttttgttaaatcagctcattttttaaccaataggccgaaatcggcaaaatcccttataaatcaaaagaatagaccgagatagggttgagtgttgttccagtttggaacaagagtccactattaaagaacgtggactccaacgtcaaagggcgaaaaaccgtctatcagggcgatggcccactacgtgaaccatcaccctaatcaagttttttggggtcgaggtgccgtaaagcactaaatcggaaccctaaagggagcccccgatttagagcttgacggggaaagccggcgaacgtggcgagaaaggaagggaagaaagcgaaaggagcgggcgctagggcgctggcaagtgtagcggtcacgctgcgcgtaaccaccacacccgccgcgcttaatgcgccgctacagggcgcgtccattcgccattcaggctgcgcaactgttgggaagggcgatcggtgcgggcctcttcgctattacgccagctggataaaggcgcgccaaacgacctaggaattggagcgacctcatgctatacctgagaaagcaacctgacctacaggaaagagttactcaagaataagaattttcgttttaaaacctaagagtcactttaaaatttgtatacacttattttttttataacttatttaataataaaaatcataaatcataagaaattcgcttatttagaagtgtcaacaacgtatctaccaacgatttgacccttttccatcttttcgtaaatttctggcaaggtagacaagccgacaaccttgattggagacttgaccaaacctctggcgaagaattgttaattaagagctcagatcttatcgtcgtcatccttgtaatccatcgatactagtgcggccgcttgttttatatttgttgtaaaaagtagataattacttccttgatgatctgtaaaaaagagaaaaagaaagcatctaagaacttgaaaaactacgaattagaaaagaccaaatatgtatttcttgcattgaccaatttatgcaagtttatatatatgtaaatgtaagtttcacgaggttctactaaactaaaccacccccttggttagaagaaaagagtgtgtgagaacaggctgttgttgtcacacgattcggacaattctgtttgaaagagagagagtaacagtacgatcgaacgaactttgctctggagatcacagtgggcatcatagcatgtggtactaaaccctttcccgccattccagaaccttcgattgcttgttacaaaacctgtgagccgtcgctaggaccttgttgtgtgacgaaattggaagctgcaatcaataggaagacaggaagtcgagcgtgtctgggttttttcagttttgttctttttgcaaacaaatcacgagcgacggtaatttctttctcgataagaggccacgtgctttatgagggtaacatcaattcaagaaggagggaaacacttcctttttctggccctgataatagtatgagggtgaagccaaaataaaggattcgcgcccaaatcggcatctttaaatgcaggtatgcgatagttcctcactctttccttactcacgagtaattcttgcaaatgcctattatgcagatgttataatatctgtgcgtcttgagttgaagtcaggaatctaaaataaaaattaaggttaataaaaagaggaaagaaaaaaaaattaatcgatttacagaaacttgcacactaaaaatacacaactaaaagcaattacagtatgggaagtcatcgacgttatctctactatagtatattatcatttctattattatcctgctcagtggtacttgcaaaacaagataagaccccattctttgaaggtacttccaggccggccgcacacaccatagcttcaaaatgtttctactccttttttactcttccagattttctcggactccgcgcatcgccgtaccacttcaaaacacccaagcacagcatactaaatttcccctctttcttcctctagggtgtcgttaattacccgtactaaaggtttggaaaagaaaaaagagaccgcctcgtttctttttcttcgtcgaaaaaggcaataaaaatttttatcacgtttctttttcttgaaaatttttttttttgatttttttctctttcgatgacctcccattgatatttaagttaataaacggtcttcaatttctcaagtttcagtttcatttttcttgttctattacaactttttttacttcttgctcattagaaagaaagcatagcaatctaatctaagttttaattacaaggatccgtaatacgactcactatagggcccgggcgtcgacatggaacagaagttgatttccgaagaagacctcgagtaagcttggtaccgcggctagctaagatccgctctaaccgaaaaggaaggagttagacaacctgaagtctaggtccctatttatttttttatagttatgttagtattaagaacgttatttatatttcaaatttttcttttttttctgtacagacgcgtgtacgcatgtaacattatactgaaaaccttgcttgagaaggttttgggacgctcgaagatcctccggatcgtttcgccggcgtttatccagctgcattaatgaatcggccaacgcgcggggagaggcggtttgcgtattgggcgctcttccgcttcctcgctcactgactcgctgcgctcggtcgttcggctgcggcgagcggtatcagctcactcaaaggcggtaatacggttatccacagaatcaggggataacgcaggaaagaacatgtgagcaaaaggccagcaaaaggccaggaaccgtaaaaaggccgcgttgctggcgtttttccataggctccgcccccctgacgagcatcacaaaaatcgacgctcaagtcagaggtggcgaaacccgacaggactataaagataccaggcgtttccccctggaagctccctcgtgcgctctcctgttccgaccctgccgcttaccggatacctgtccgcctttctcccttcgggaagcgtggcgctttctcatagctcacgctgtaggtatctcagttcggtgtaggtcgttcgctccaagctgggctgtgtgcacgaaccccccgttcagcccgaccgctgcgccttatccggtaactatcgtcttgagtccaacccggtaagacacgacttatcgccactggcagcagccactggtaacaggattagcagagcgaggtatgtaggcggtgctacagagttcttgaagtggtggcctaactacggctacactagaaggacagtatttggtatctgcgctctgctgaagccagttaccttcggaaaaagagttggtagctcttgatccggcaaacaaaccaccgctggtagcggtggtttttttgtttgcaagcagcagattacgcgcagaaaaaaaggatctcaagaagatcctttgatcttttctacggggtctgacgctcagtggaacgaaaactcacgttaagggattttggtcatgagattatcaaaaaggatcttcacctagatccttttaaattaaaaatgaagttttaaatcaatctaaagtatatatgagtaaacttggtctgacagttaccaatgcttaatcagtgaggcacctatctcagcgatctgtctatttcgttcatccatagttgcctgactccccgtcgtgtagataactacgatacgggagggcttaccatctggccccagtgctgcaatgataccgcgagacccacgctcaccggctccagatttatcagcaataaaccagccagccggaagggccgagcgcagaagtggtcctgcaactttatccgcctccatccagtctattaattgttgccgggaagctagagtaagtagttcgccagttaatagtttgcgcaacgttgttgccattgctacaggcatcgtggtgtcacgctcgtcgtttggtatggcttcattcagctccggttcccaacgatcaaggcgagttacatgatcccccatgttgtgcaaaaaagcggttagctccttcggtcctccgatcgttgtcagaagtaagttggccgcagtgttatcactcatggttatggcagcactgcataattctcttactgtcatgccatccgtaagatgcttttctgtgactggtgagtactcaaccaagtcattctgagaatagtgtatgcggcgaccgagttgctcttgcccggcgtcaatacgggataataccgcgccacatagcagaactttaaaagtgctcatcattggaaaacgttcttcggggcgaaaactctcaaggatcttaccgctgttgagatccagttcgatgtaacccactcgtgcacccaactgatcttcagcatcttttactttcaccagcgtttctgggtgagcaaaaacaggaaggcaaaatgccgcaaaaaagggaataagggcgacacggaaatgttgaatactcatactcttcctttttcaatattattgaagcatttatcagggttattgtctcatgagcggatacatatttgaatgtatttagaaaaataaacaaataggggttccgcgcacatttccccgaaaagtgccacctgaacgaagcatctgtgcttcattttgtagaacaaaaatgcaacgcgagagcgctaatttttcaaacaaagaatctgagctgcatttttacagaacagaaatgcaacgcgaaagcgctattttaccaacgaagaatctgtgcttcatttttgtaaaacaaaaatgcaacgcgagagcgctaatttttcaaacaaagaatctgagctgcatttttacagaacagaaatgcaacgcgagagcgctattttaccaacaaagaatctatacttcttttttgttctacaaaaatgcatcccgagagcgctatttttctaacaaagcatcttagattactttttttctcctttgtgcgctctataatgcagtctcttgataactttttgcactgtaggtccgttaaggttagaagaaggctactttggtgtctattttctcttccataaaaaaagcctgactccacttcccgcgtttactgattactagcgaagctgcgggtgcattttttcaagataaaggcatccccgattatattctataccgatgtggattgcgcatactttgtgaacagaaagtgatagcgttgatgattcttcattggtcagaaaattatgaacggtttcttctattttgtctctatatactacgtataggaaatgtttacattttcgtattgttttcgattcactctatgaatagttcttactacaatttttttgtctaaagagtaatactagagataaacataaaaaatgtagaggtcgagtttagatgcaagttcaaggagcgaaaggtggatgggtaggttatatagggatatagcacagagatatatagcaaagagatacttttgagcaatgtttgtggaagcggtattcgcaatattttagtagctcgttacagtccggtgcgtttttggttttttgaaagtgcgtcttcagagcgcttttggttttcaaaagcgctctgaagttcctatactttctagagaataggaacttcggaataggaacttcaaagcgtttccgaaaacgagcgcttccgaaaatgcaacgcgagctgcgcacatacagctcactgttcacgtcgcacctatatctgcgtgttgcctgtatatatatatacatgagaagaacggcatagtgcgtgtttatgcttaaatgcgtacttatatgcgtctatttatgtaggatgaaaggtagtctagtacctcctgtgatattatcccattccatgcggggtatcgtatgcttccttcagcactaccctttagctgttctatatgctgccactcctcaattggattagtctcatccttcaatgctatcatttcctttgatattggatcatactaagaaaccattattatcatgacattaacctataaaaataggcgtatcacgaggccctttcgtc | ^2^ |

1. Varemo L, Nielsen J, Nookaew I. Enriching the gene set analysis of genome-wide data by incorporating directionality of gene expression and combining statistical hypotheses and methods. Nucleic Acids Res. 2013;41(8):4378-91.

2. Partow S, Siewers V, Bjorn S, Nielsen J, Maury J. Characterization of different promoters for designing a new expression vector in *Saccharomyces cerevisiae*. Yeast. 2010;27(11):955-64.
